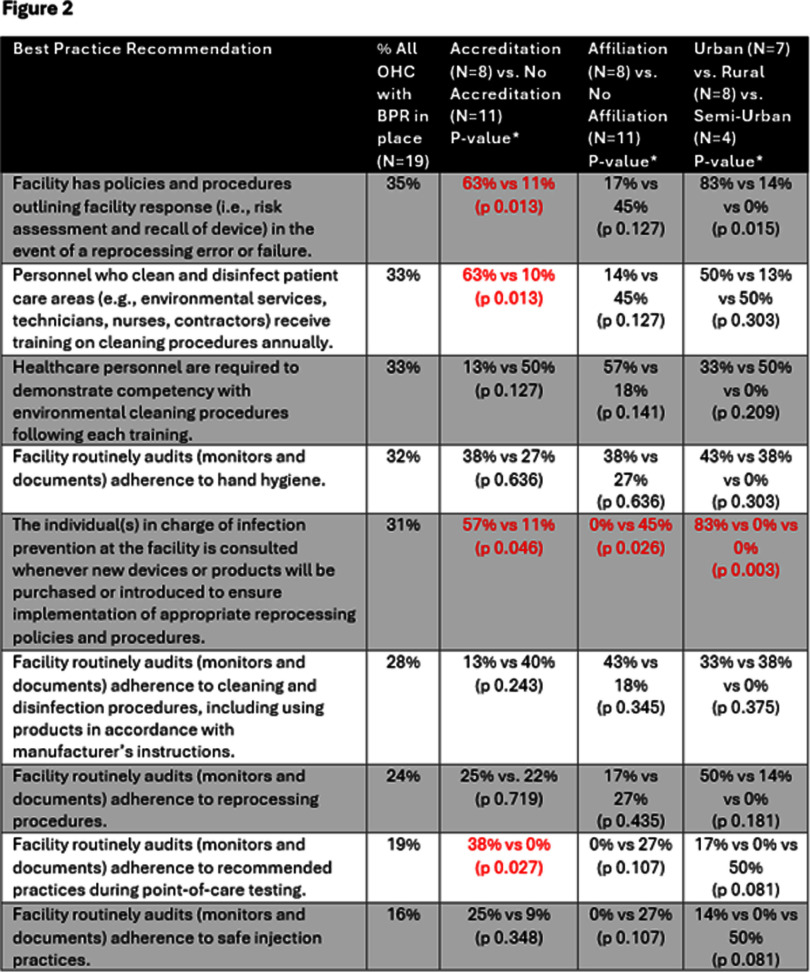# Infection Prevention Program Infrastructure and Implementation of Best Practice Recommendations in Outpatient Healthcare Facilities

**DOI:** 10.1017/ash.2025.385

**Published:** 2025-09-24

**Authors:** Kate Tyner, Jody Scebold, M. Salman Ashraf, Dan German, Rebecca Martinez, Josette McConville, Mounica Soma, Juan Teran Plasencia

**Affiliations:** 1 Nebraska ICAP, Nebraska Medicine; 2 Nebraska Medical Center; 3 University of Nebraska Medical Center; 4 Nebraska Medicine; 5 Nebraska Medicine; 6 Nebraska ICAP/Nebraska Medicine; 7 Nebraska Medicine; 8 UNMC

## Abstract

**Background:** Nebraska (NE) Infection Control Assessment and Promotion Program (ICAP) is supported by the Nebraska DHHS Healthcare-Associated Infection (HAI) program via a CDC grant and works to assess and improve infection prevention and control (IPC) programs in all types of healthcare facilities. CDC recommends that outpatient healthcare facilities (OHFs) develop and maintain IPC programs; however, littleis known about the infrastructure of IPC programs in OHFs. NE-ICAP performed onsite assessments to review the implementation of best practice recommendations (BPRs) in these programs. **Method:** Onsite IPC assessments were conducted in OHFs from January 2020 to February 2024. The assessment questions were based primarily on the CDC 2016 Infection Control Assessment and Response (ICAR) tool, complemented by the CMS Hospital Infection Control Worksheet. Assessments included interviews and onsite observations. A total of 66 BPRs were assessed for implementation. Descriptive statistics were calculated using Microsoft Excel for assessment responses and demographic information. BPRs were classified based on hospital affiliation, accreditation status (based on certification by recognized accrediting bodies), and urban-rural designation (based on USDA rural-urban commuting area codes). The chi-square test for independence was performed in SPSS 20 to assess for statistically significant differences across these categories using a threshold of p < 0.05. **Result:** A total of 19 OHFs had onsite assessments. 42.1% had external accreditation, 77.8% had at least one individual trained in infection prevention regularly available, and 36.8% were considered urban (figure 1). Domains with the lowest compliance (percentage of BPRs in place) included injection safety (48.8%), device reprocessing (49.7%), and personal protective equipment (51.8%). Notable BPRs associated with less than 35% compliance are listed in figure 2. Accredited facilities demonstrated greater compliance with BPRs related to device reprocessing. **Conclusion:** Important IPC gaps exist in OHFs. Onsite assessments are crucial for evaluating IPC program infrastructure and highlighting areas for improvement. Further studies are needed to understand why accreditation is associated with better compliance with BPRs and the factors contributing to its success.

**Figure:**
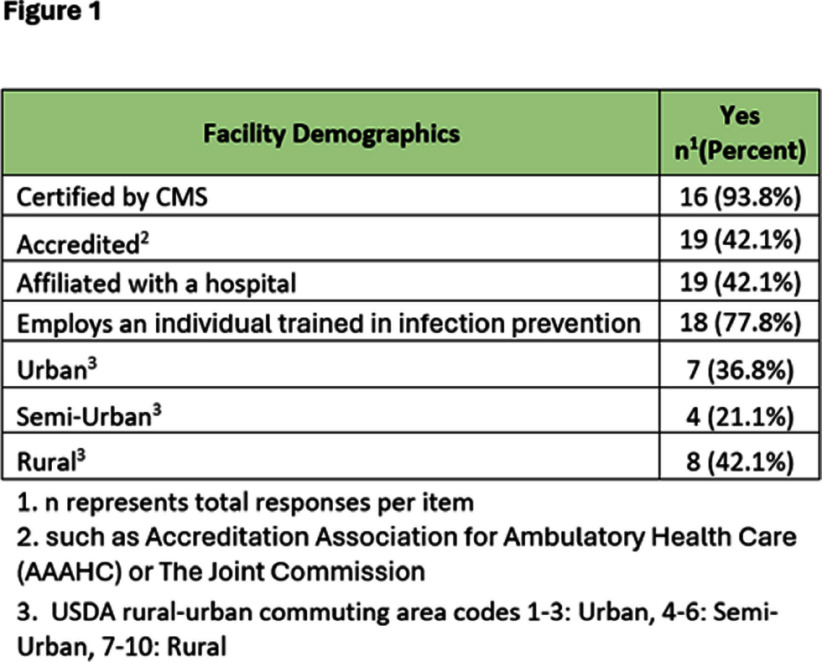

**Figure:**